# Unveiling patterns in clinical data: exploring the role of large language models and clustering algorithms

**DOI:** 10.3389/frai.2026.1737530

**Published:** 2026-03-09

**Authors:** Abbas S. Ali, Subi Gandhi, Syed H. Jafri, Mohammed M. Ali, Syed Y. Raza, Sulaiman Samian, James Mehaffey

**Affiliations:** 1 Department of Medicine, Division of Cardiology, Kern Medical, Bakersfield, CA, United States; 2 Center for Rural Resilience, Tarleton State University, Stephenville, TX, United States; 3 Department of Accounting, Finance and Economics, Tarleton State University, Stephenville, TX, United States; 4 Alumni, West Virginia University, Morgantown, WV, United States; 5 Burnett School of Biomedical Sciences, College of Medicine, University of Central Florida, Orlando, FL, United States; 6 Department of Cardiology, Heart and Vascular Institute, West Virginia University, Morgantown, WV, United States

**Keywords:** endocarditis, large language models, medical informatics, natural language processing, precision medicine, predictive Modeling

## Abstract

**Objective:**

Large Language Models (LLMs) have shown exceptional performance in natural language processing, yet their utility in structured clinical data analysis remains relatively underexplored. This pilot study investigates whether LLM-generated embeddings can preserve the structural integrity of clinical datasets and enhance predictive modeling, particularly in resource-constrained settings.

**Methods:**

We applied dimensionality reduction techniques such as Principal Component Analysis (PCA), t-distributed Stochastic Neighbor Embedding (t-SNE), and k-means clustering to compare original data structures with those derived from LLM embeddings. Evaluation metrics included cosine similarity, area under the curve (AUC), and *R*^2^, applied across 100 synthetic datasets and two real-world clinical datasets: the UCI medical database and endocarditis patient records. We assessed multiple LLM architectures, including BERT, RoBERTa, Llama 2, and E5-small, focusing on predictive accuracy and computational efficiency.

**Results:**

LLM embeddings closely mirrored original data structures, with BERT achieving a cosine similarity of 0.95 on linear datasets and Llama 2 (30B) reaching 0.85 on quadratic datasets, albeit with higher computational costs. Predictive performance improved significantly across the board with increases in subject variable ratio (SVR), three groups were identified similar performance, assisted better and assisted significantly better. These groups differed based upon the equation used to generate synthetic data.

**Discussion:**

These findings highlight the potential of LLMs to enhance structured data analysis by identifying optimal conditions, such as SVR thresholds, for their practical use. The trade-off between computational cost and performance across different LLM architectures is also emphasized, suggesting the need for context-specific model selection.

**Conclusion:**

LLMs can be effectively leveraged to repurpose existing clinical datasets for individualized clinical questions, such as optimizing surgical timing for patients with infective endocarditis and embolic stroke. This approach advances precision medicine and supports data-driven clinical decision-making.

## 1 Introduction

The integration of artificial intelligence (AI) into healthcare is transforming clinical research, with large language models (LLMs) such as ChatGPT playing a pivotal role (Alum and Ugwu, 2025; Parekh et al., 2023; Nzeako et al., 2025; Ali et al., 2023). These models offer powerful tools for interpreting complex clinical data and uncovering nuanced patterns. However, their application in healthcare is tempered by known limitations, including issues with accuracy, contextual relevance, and hallucination (Li et al., 2023; Shah et al., 2024). To mitigate these risks, effective prompt engineering, setting the temperature of the LLM and strategic querying are essential, along with rigorous evaluation of LLM outputs against domain expertise to ensure reliability (Shah et al., 2024).

Traditionally, researchers have used statistical methods such as univariate analysis, stepwise regression, and multivariate analysis to identify and evaluate cardiovascular disease risk factors. These approaches have been instrumental in uncovering associations, such as the link between high cholesterol and heart disease. With the advent of greater computational power, machine learning (ML) methods, both supervised and unsupervised, have enabled the analysis of high-dimensional datasets, revealing hidden patterns (Nadif and Role, 2021; Tirthyani et al., 2024; Vinci, 2024). However, ML models often lack transparency, which makes their decision-making processes challenging to interpret (Golovin, 2024; Patil, 2024).

To address the challenge of interpreting complex data, dimensionality reduction techniques such as Principal Component Analysis (PCA) and t-distributed Stochastic Neighbor Embedding (t-SNE) are commonly used (Ray et al., 2021). PCA projects data into lower dimensions by capturing the greatest variance through eigenvectors, typically highlighting linear relationships. In contrast, t-SNE excels at revealing non-linear patterns and local clusters by mapping data into a probabilistic space and is commonly used for visualization. Although t-SNE does not preserve global structure and can be computationally intensive, it is often preceded by PCA to improve efficiency and clustering quality (Shah and Silwal, 2019; Song et al., 2013).

**Figure 1:**
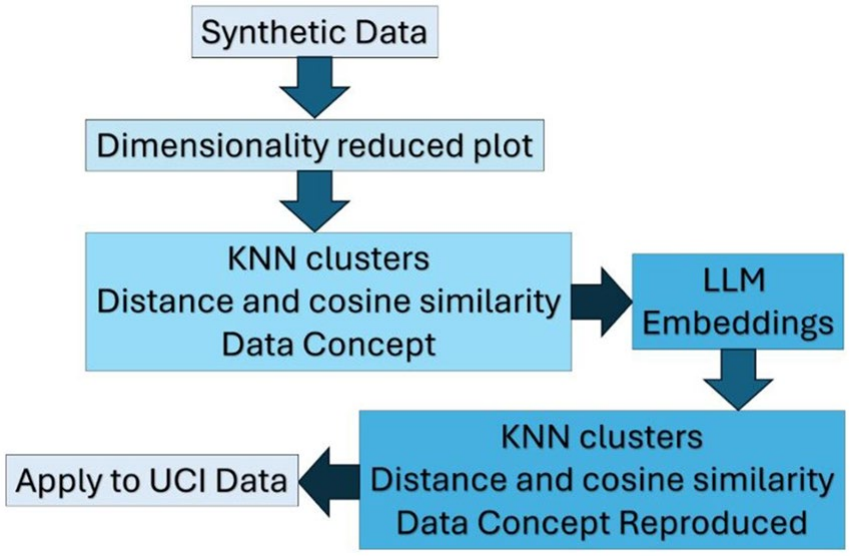
Comprehensive framework for evaluating LLM-assisted analysis.

In addition to dimensionality reduction, pattern recognition techniques like K-Nearest Neighbors (KNN) and k-means clustering are widely used (Zriaa and Amali, 2021). KNN is a simple yet effective classification method that identifies the ‘k’ closest data points to a query point and uses them to determine its class. It is often confused with k-means clustering, which instead relies on centroids and iterative repositioning to form clusters. Unlike k-means, KNN does not involve centroids or iterative updates and is purely distance-based (Cunningham and Delany, 2020) (Figure 1).

Modern large language models (LLMs), such as OpenAI’s GPT series, are built on the Transformer architecture introduced by Vaswani et al. (2017) (Yenduri et al., 2024). These models generate coherent and contextually relevant text by predicting the next word in a sequence based on prior context (Alomari et al., 2023; Chafi et al., 2023). Central to their design is the self-attention mechanism, which captures global dependencies and enables parallel processing for improved scalability (Ahmed et al., 2023). A key innovation—multi-head attention—allows multiple attention heads to operate simultaneously, capturing diverse linguistic features including syntactic and semantic relationships. These outputs are concatenated and transformed to produce rich, high-dimensional embeddings that encode complex semantic relationships. Variations in training data, tokenization, attention mechanisms, and directionality contribute to differences among models (Passi et al., 2024; Devlin et al., 2018; Raiaan et al., 2024).

In healthcare, Transformer-based models have demonstrated potential in uncovering hidden determinants, supporting personalized care, and enhancing predictive modeling (Alum and Ugwu, 2025). This study leverages embeddings from advanced LLMs to represent tabular patient data as natural language, withholding outcome variables to preserve analytic integrity. Structured clinical data often suffers from sparsity and multicollinearity, particularly with high-dimensional categorical variables, where traditional preprocessing methods like one-hot encoding can inflate feature space and reduce interpretability (Zhao et al., 2017).

By modeling inter-variable relationships through attention mechanisms, we aimed to reduce preprocessing, preserve semantic structure, and improve both accuracy and interpretability. Specifically, we aimed to evaluate the feasibility of LLMs in structured clinical data analysis by addressing sparsity and multicollinearity, modeling complex relationships, assessing embedding integrity via clustering metrics, identifying optimal performance conditions, and supporting precision medicine through interpretable insights.

## 2 Methods

### 2.1 Comparative evaluation framework for transformer-based embedding models

#### 2.1.1 Selection of transformer architectures and preprocessing pipeline

We evaluated Transformer models including BERT, RoBERTa, DistilBERT, ERNIE, T5, XLNet, GatorTron, MiniLM, E5-small-cluster, and LLaMA2-30B, selected for computational feasibility, architectural diversity, and performance across varied data relationships (Devlin et al., 2018; Raiaan et al., 2024; Zhang et al., 2019; Raffel et al., 2019; Yang et al., 2019; Yang et al., 2022; Wang et al., 2020; Wang et al., 2022; Shindo et al., 2025)Performance was assessed using geometric (centroid distances, cosine similarity) and predictive metrics (*R*^2^, AUC), with ROC-based AUC for kNN cluster prediction mitigating high-dimensional degradation (Salem et al., 2025; Aggarwal et al., 2001; Saito and Rehmsmeier, 2015). Embedding time and comparisons with traditional ML models were recorded, using *R*^2^, AUC, or MAE depending on outcome type. Hyperparameters were tuned via 5-fold Grid Search Cross-Validation.

**Figure 2:**
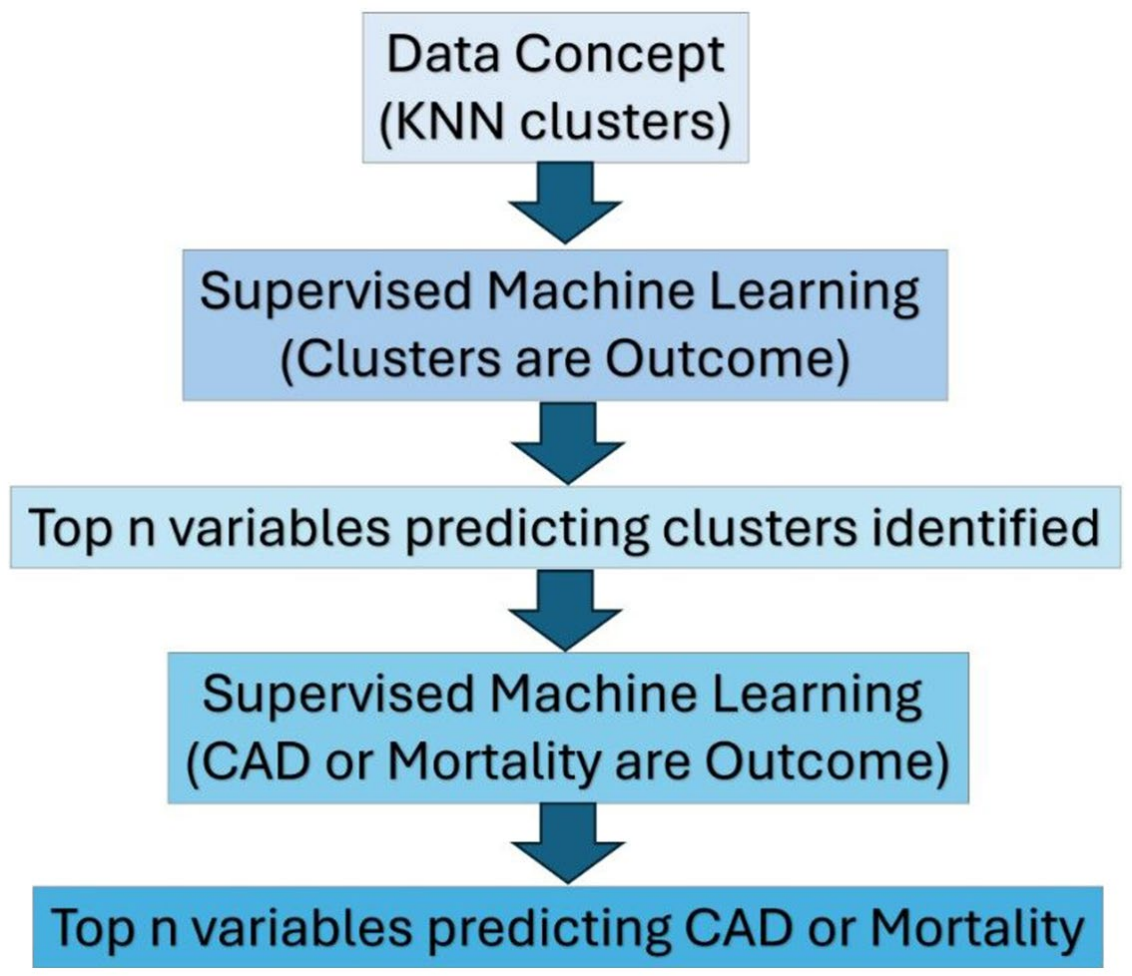
Steps involving integration of LLM, clustering, and ML techniques.

Preprocessing included low-variance feature removal (Figure 2), median/mode imputation, and one-hot encoding. SMOTE was applied post–cross-validation to address class imbalance. An ensemble Voting Classifier (Random Forest, XGBoost, SVM) was used, with hyperparameters tuned to prioritize precision and recall over accuracy for balanced clinical performance.

#### 2.1.2 Clustering methodology and embedding comparison

To analyze high-dimensional data, K-means clustering was applied using Euclidean distance and cosine similarity (Uhryn and Kalancha, 2024; Mussabayev, 2024). Given the limitations of these metrics in high-dimensional spaces (Zahariah and Midi, 2024), PCA and t-SNE were used to preserve global and local data structures, respectively. Cluster relationships were assessed using cosine similarity, Euclidean distance, and Spearman correlation to evaluate how well LLM-generated embeddings preserved original data geometry. Optimal cluster counts were determined via mean pooling, elbow method, and silhouette scores. Feature selection improved clustering coherence, as evidenced by higher silhouette scores, which validated the approach.

#### 2.1.3 Dataset design, SVR analysis, and model performance

We generated 100 synthetic datasets (500 rows each) with SVRs ranging from 10 to 100, incorporating exponential, cubic, quadratic, and linear relationships. These datasets included continuous and categorical variables with embedded collinearity, allowing us to assess how SVR and variable types influence model performance (Supplementary Figure S1).

**Figure 3:**
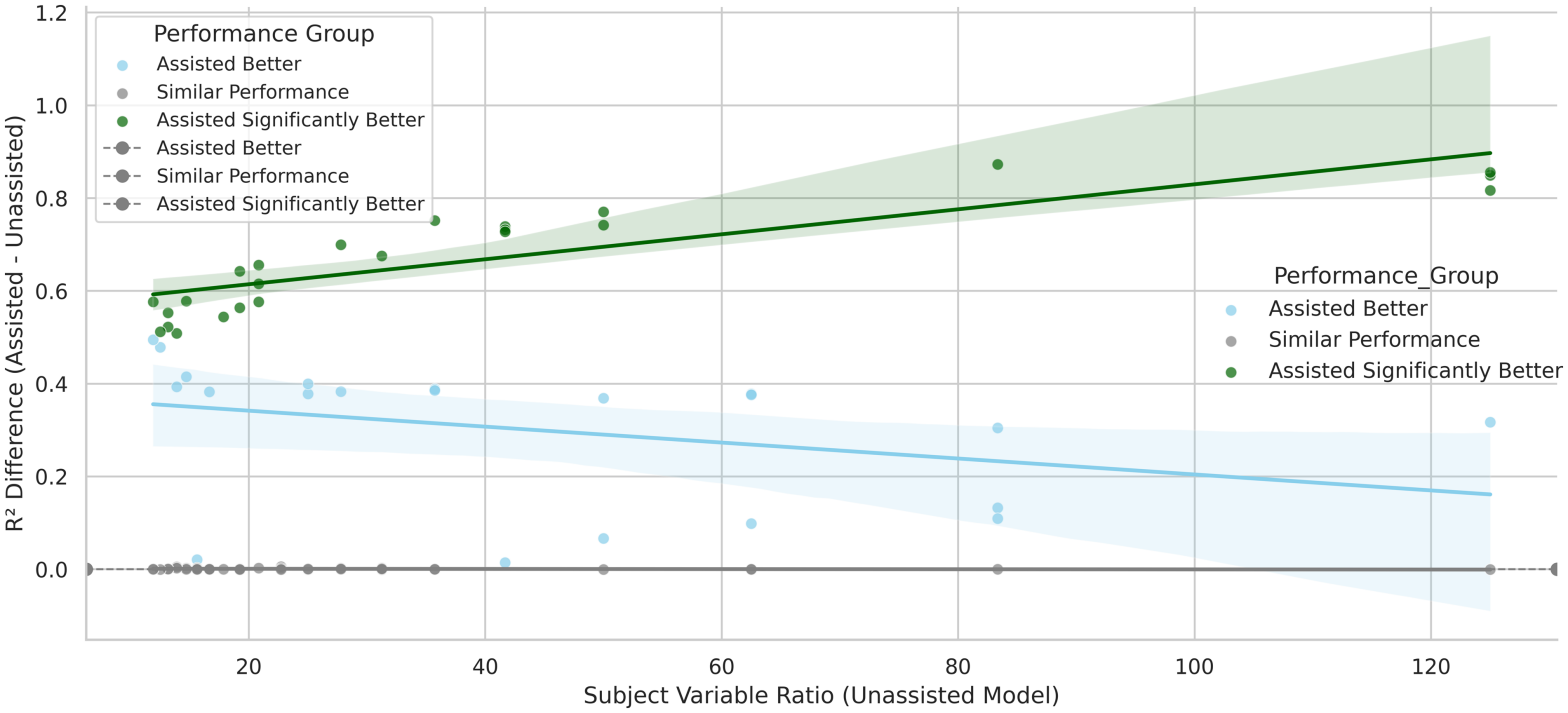
Illustration of mean *R*^2^ and 95% CI across linear models: Optimal LLM performance at SVR 35–40 and 15–20 categorical variables.

Model performance was evaluated using linear regression, random forest, and gradient boosting, with metrics including *R*^2^, RMSE, MAE, and AUC (Supplementary Figures S2, S3, and Figure 3). LLM-assisted models using eight embeddings outperformed unassisted models in 79% of binary tasks. SHAP scores identified top predictors, and paired t-tests confirmed significance. Fisher’s Exact Test (*p* = 0.0001; Supplementary Table S1) showed a strong association between model performance and dataset type. While linear and cubic datasets showed similar results across models, exponential and quadratic datasets benefited most from LLM assistance—none favored unassisted models.

#### 2.1.4 Categorical complexity and predictive reliability

Supplementary Figure S4 shows that lower SVRs (<20) and high categorical complexity (>20 variables) reduce model reliability. Residual plots and the Breusch-Pagan test (Supplementary Figure S4) revealed heteroscedasticity at low SVRs. LLM assisted models performed better than LLM unassisted models with fewer subjects and higher number of variables (Figure 3).

#### 2.1.5 Embedding fidelity and concept capture

**Figure 4:**
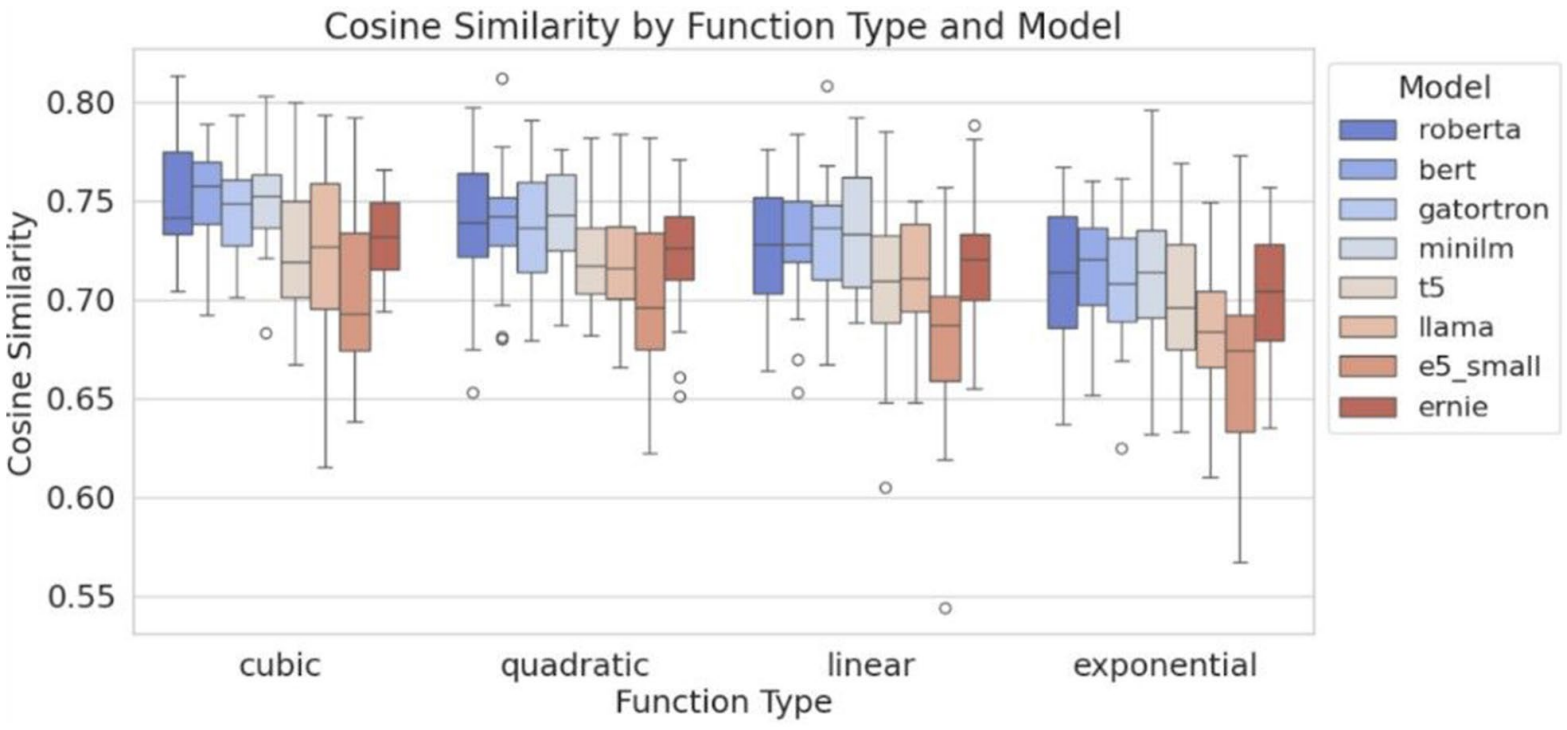
Boxplot of cosine similarity scores.

**Figure 5:**
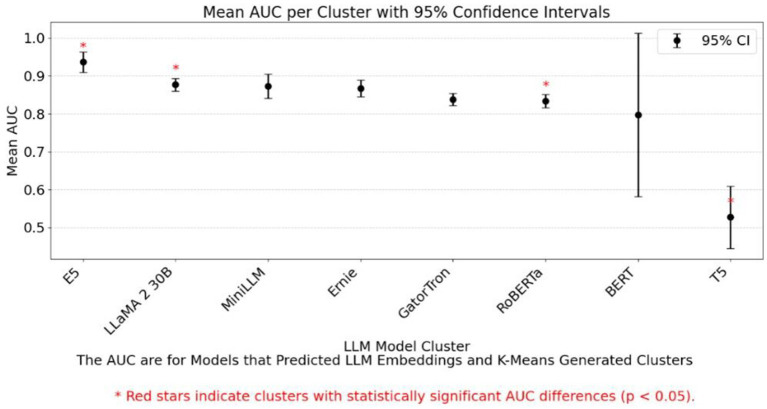
Comparison of mean AUC scores: LLM embeddings vs. K-means clusters for binary outcome (median split).

We assessed whether LLM embeddings preserved original data structure using cosine similarity (Supplementary Figures S6–8 and Figure 4). BERT achieved the highest similarity (0.95), followed by E5 and LLaMA 2 30B. Ensemble models performed best in high-quality clusters, while logistic regression excelled in RoBERTa-derived clusters but struggled with lower-performing embeddings like T5. Supplementary Figure S6 illustrates the reconstruction of the original outcome variable used to generate the synthetic datasets. When cluster assignments derived from different LLMs were incorporated as predictors, the resulting AUC values varied across supervised ML algorithms, reflecting differences in how each model leveraged the LLM-based cluster structure. Supplementary Figures S9 and Figure 5 show confidence intervals for cosine similarity and AUC, reinforcing the superior performance of BERT and E5-small.

#### 2.1.6 Feature selection and SVR balance

Feature selection improved generalizability by reducing dimensionality and overfitting. Maintaining SVRs above 20 and limiting categorical variables to fewer than 20 enhanced model accuracy and interpretability (Figure 3).

### 2.2 Clinical variable context

#### 2.2.1 Categories of clinical data

This study utilized clinical data spanning key categories for predictive modeling, including demographics (age, gender, socioeconomic status) from the UCI dataset of 76 variables across 303 patients. Medical history (e.g., hypertension, diabetes, smoking), lab values (blood sugar, cholesterol), and diagnostic data (ECG abnormalities, ST depression, thallium stress test results) were included to assess cardiac risk and disease severity. Each variable was selected for its relevance to improving model accuracy and informing patient prognosis.

#### 2.2.2 Relevance to clinical prediction

Variables included in the clinical prediction models were selected for their relevance and impact on predictive accuracy. By incorporating demographics, medical history, lab results, and diagnostic findings, the models were designed to reflect real-world clinical decision-making. This careful selection enhanced both model interpretability and clinical relevance.

#### 2.2.3 Variable processing and missing data

The endocarditis dataset variables were grouped into three types—continuous, categorical, and ordinal—each contributing uniquely to predictive modeling. Continuous variables (e.g., BMI, age, EF, troponin) offered quantifiable health indicators. Categorical variables (e.g., valve type, pulmonary dysfunction, IV drug use, stroke) captured discrete clinical traits for risk stratification. Ordinal variables (e.g., valvular insufficiency severity, surgical urgency, vegetation size) reflected ranked assessments of disease severity and intervention urgency. To manage missing data, median imputation was used for continuous variables and mode imputation for categorical ones (Occhipinti, 2024; Memon et al., 2023). To address class imbalance—especially for rare outcomes like mortality, the Synthetic Minority Oversampling Technique (SMOTE) was applied after cross-validation (Dou et al., 2024; Elreedy and Atiya, 2019), improving model robustness. A summary of preprocessing steps and their purposes is provided in Table 1.

**Table 1:** Summary of steps, techniques, and purpose.

Step	Technique	Purpose
Handling missing data	Multiple imputation	Fill missing values (continuous variables)
Numeric preprocessing	Standard scaler	Rescale variables to zero mean/unit variance
Categorical preprocessing	One-hot encoding	Convert categorical variables to binary features
Data balancing	SMOTE	Generates synthetic samples to balance imbalanced datasets and improve model performance.

#### 2.2.4 Sentence construction as a bridge between structured data and LLMs

Structured data rows—excluding outcome variables—were converted into natural language sentences using a Python script (see Supplemental Folder and GitHub repository: https://github.com/aliabbasmd/response_reviewer). These were processed by LLMs to generate embeddings, which were clustered via k-means. Cluster assignments served as categorical features in LLM-assisted models, while unassisted models omitted this step. In clinical datasets, clusters were treated as intermediate outcomes; SHAP analysis identified key predictors of cluster membership, informing a refined model for the primary outcome. This two-step approach enhanced both performance and interpretability.

### 2.3 Resource and scalability considerations

#### 2.3.1 Resource requirements and scalability challenges

LLMs, though optimized for text (Bommasani et al., 2021), can still reveal model behavior through embeddings, even with proprietary weights (Lundberg and Lee, 2017). We used SHAP and LIME to assess alignment with clinical judgment in complex cases like endocarditis with stroke (Lundberg and Lee, 2017; Ribeiro et al., 2016; Bender et al., 2021). Interpretability is essential for safe decision-making.

LLMs require GPU-based parallel processing, with deployment dependent on tools like CUDA, Torch, and Hugging Face (Moore, 2023). Quantized models reduce memory requirements and improve accessibility, but do so at the cost of some loss in numerical precision and model accuracy. In contrast, full-precision (non-quantized) models demand extremely large amounts of VRAM to perform high-precision calculations. These requirements frequently exceed available hardware capacity, causing systems to crash or terminate processes due to ‘memory explosion.’ Furthermore, their extreme sensitivity allows them to perfectly memorize data noise, causing the loss function to collapse to zero through overfitting rather than true learning. Frequent software updates and infrastructure demand challenge reproducibility, highlighting the need for institutional support and skilled, prompt engineers to ensure scalable, impactful use.

#### 2.3.2 Description of datasets

Three datasets were used to evaluate the performance of LLM-assisted models in structured clinical data analysis. Table 2 summarizes their characteristics and rationale. The first two datasets supported conceptual development, while the third focused on real-world clinical application.

**Table 2:** Characteristics of the three datasets used in the study.

Number of dataset	Number of rows	Number of variables	Non-zero data entries (%)
Synthetic data	500	2 random continuous, 1 calculated, 2 to 40 categorical	100
UCI data	303	13 (7 categorical)	91.6
West Virginia University ‘Raw’ Endo-caritas Data (de-identified data)	442	58 (51 categorical)	15

##### 2.3.2.1 Synthetic data

To assess the sensitivity and robustness of our analytical pipeline to variations in data structure, we generated 100 synthetic datasets, each containing 500 observations. Within these datasets, we systematically manipulated the ratio of samples to variables (500 samples and 2–40 categorical variables) to represent settings with differing levels of dimensional complexity. We introduced controlled multicollinearity by allowing selected categorical predictors to exert direct influence on the outcome variable. This experimental design enabled us to evaluate pipeline performance across a broad spectrum of feature dependencies, redundancy patterns, and structural complexities that often characterize real-world clinical data.

By simulating such diverse conditions, we were able to rigorously interrogate the stability and reliability of our natural language processing (NLP) workflow. In particular, this approach allowed us to test whether the model retained consistent behavior under challenging scenarios including high feature overlap, dominance of specific categorical attributes, and varying degrees of noise. As various LLMs have diverse training data and methods our process also teased out the particular kind of LLM best for a particular data structure. Collectively, these experiments provided a controlled environment to evaluate whether the pipeline remained robust when confronted with data characteristics known to degrade model interpretability and clustering performance (Supplementary Table S1).

Each row of every synthetic dataset was transformed into a natural-language sentence using a custom text-generation function. The resulting sentences were stored in a dedicated “combined” column, creating a structured text corpus in which each row served as an independent qualitative unit. To convert these sentences into numerical representations suitable for downstream analysis, we generated text embeddings using the final hidden state of the [CLS] token from multiple Transformer architectures. Both general-purpose models (RoBERTa, DistilBERT) and domain-specific biomedical models (e.g., GatorTron) were employed to capture theoretical variability in semantic representation. Larger models were processed using batch-based inference to accommodate memory constraints, resulting in a final embedding matrix for each model type.

Clustering analysis was performed using K-means to evaluate how effectively each model’s embeddings grouped semantically similar entries. The optimal number of clusters was determined using the Elbow Method, which identifies the inflection point at which additional clusters yield diminishing returns in explained variance. Cluster quality and cohesion were then assessed using established internal validation metrics, including the Silhouette Score and the Calinski–Harabasz Index. These metrics allowed systematic comparison of embedding quality across models and informed selection of the most effective representation for subsequent analysis.

To further characterize cluster separability and global embedding geometry, high-dimensional embeddings were visualized using a two-stage dimensionality reduction approach. Principal Component Analysis (PCA) was first applied to preserve maximal variance while reducing dimensionality. Subsequently, t-Distributed Stochastic Neighbor Embedding (t-SNE) was used to project the data into two dimensions. This combined approach provided a qualitative assessment of the semantic structure of the embeddings and facilitated visual inspection of cluster boundaries, local coherence, and overall geometric organization.

The project’s GitHub repository contains not only the primary analysis scripts but also detailed, step-by-step walkthroughs located in the [GitHub] and Ahmed et al. (2023). These examples illustrate the full workflow for generating T5-based text embeddings and conducting unsupervised clustering to identify groups of clinical phenotypes. This supplemental material provides users with concrete demonstrations of the modeling process, facilitating hands-on exploration and replication.

A streamlined *README.md* file integrates these components and provides clear guidance on repository structure, dependencies, and execution steps. Executing the python files sequentially allows for reproduction and insight into the synthetic data. Worked out UCI examples illustrate clinical insights. Together, these resources support transparency, reproducibility, and knowledge transfer, making the workflow accessible for both research replication and educational use in clinical informatics and training environments.

##### 2.3.2.2 West Virginia University endocarditis data

We used de-identified clinical data from patients with endocarditis (Supplementary Table S2) who had undergone life-saving heart valve surgery (number of variables = 517; sample size = 442), with a subject-to-variable ratio of 0.85 in order to implement and assess our technique (Figure 3). The state of West Virginia is plagued by illicit drug use (Merino et al., 2019; Bhandari et al., 2022), and the data set was representative of this population in the hospital data. Life-threatening infections such as IE are on the rise in West Virginia (WV) in conjunction with the injection drug use epidemic (Raiaan et al., 2024; Zhao et al., 2017).

We followed the steps outlined in section 2.3.2.1 and used the same transformers to identify the underlying clinical concepts for the WVU IE clinical data. Figures 3 and 4 illustrate the process of analyzing the WVU-HVI data and its characteristics.

The West Virginia University Endocarditis dataset cannot be publicly released due to privacy and confidentiality restrictions associated with the underlying clinical data and can be made available on request.

##### 2.3.2.3 University of California Irvine data

In this step, we used the University of California Irvine (UCI) clinical data repository (Ahmed et al., 2023), which is a robust dataset for CAD, a leading cause of mortality in the United States (Passi et al., 2024). Data from this repository (Supplementary Table S2) were used to assess whether LLM-assisted ML identifies features associated with the outcome variable similar to conventional ML. Using this repository, we demonstrated nine perspectives on CAD data using various LLM transformers, including Llama2, BERT, RoBERTa, DistilBERT, T5, Ernie, Gatorton, and GatortonS, and XLNet.

A similar analysis involving dimensionality reduction techniques was performed using UCI data (Devlin et al., 2018). The data and Python code available on their website were downloaded and processed. This analysis aimed to identify the most significant features in the data that contribute to the clustering of the embeddings. Because directly obtaining this information from t-SNE is not feasible, we used a different clustering technique, namely K-means. The K-means-generated clusters were subsequently superimposed onto the t-SNE clusters to evaluate their similarity. Next, we thoroughly assessed the features associated with the K-means clusters using ML techniques to identify a list of refined variables. Subsequently, we used these variables to construct supervised ML models to predict the outcome of interest. The UCI dataset, a widely published resource, included clinical and diagnostic features such as stress test results and coronary angiography, serving as a benchmark for comparing variable distributions in confirmed versus unconfirmed coronary artery disease cases (Supplementary Table S3).

The Python code used to run the analyses is publicly available on GitHub (link above).

None of the synthetic data used in this study were part of any language model’s pretraining corpus, especially the synthetic and endocarditis data.

LLM-assisted and Random Forest models showed similar performance in predicting coronary artery disease (AUCs of 0.88 and 0.89). The LLM model prioritized heart rate, ST depression, age, cholesterol, and blood pressure, while Random Forest emphasized thallium imaging and exercise-induced angina. These results align with SVR and categorical variable counts, confirming both models performed similarly under these conditions.

## 3 Results

### 3.1 Performance metrics

Cosine similarity was used to assess how well LLM embeddings preserved the structure of the original datasets. Despite requiring 24 h to process 100 datasets, LLaMA 2 30B underperformed compared to smaller, faster models like BERT and E5-small, which completed the same task in minutes. Table 3 summarizes the trade-off between performance (cosine similarity) and resource utilization (processing time), highlighting the efficiency of smaller models.

**Table 3:** Performance-resource tradeoff assessment.

Name of LLM	Cosine distance for data generated using exponential equations (Mean ± Confidence Interval)	Processing time (min) for 100 Files
bert	0.716 ± 0.012	Less than 5 min
minillm	0.715 ± 0.014	Around 30 min
roberta	0.711 ± 0.015	Less than 5 min
gatortron	0.707 ± 0.013	Less than 5 min
ernie	0.703 ± 0.014	Less than 5 min
t5	0.700 ± 0,015	Less than 5 min
Llama 2 30b	0.684 ± 0.016	24 h (1,440 min)
T5 small	0.665 ± 0.021	Less than 5 min

### 3.2 Comparative evaluation against established methods

Supplementary Figures S2, and Figure 3 illustrate scenarios where LLM-assisted models outperform traditional models, particularly under favorable SVR and categorical complexity conditions. Supplementary Figure S7 shows that specific LLMs perform better depending on the underlying data-generating function. For example, Supplementary Figure S8 demonstrates that BERT significantly outperforms E5-small for exponential functions, while Figure 5 highlights AUC differences across models, *p* < 0.05 marked by red stars.

#### 3.2.1 Statistical validation of findings

To validate robustness, we ran multiple iterations across LLMs, modeling each LLM-derived cluster as the target (Y) and using other variables as predictors (X). SHAP analysis identified the top 20 features, with Random Forest outperforming Gradient Boosting in efficiency and consistency. AUC values remained stable, and the top 10 SHAP features were consistent across runs—suggesting LLM embeddings did not add predictive value beyond structured features. If deeper latent patterns were captured, we would expect distinct SHAP profiles or improved AUCs. However, the consistency across LLM-assisted and unassisted models suggests embeddings may effectively capture the same core concepts. Variables such as age, BMI, ejection fraction, embolism, and cardiac arrest consistently ranked as top predictors of hospital mortality. Notably, “Days from stroke to surgery” emerged as a key variable (Supplementary Figure S9), with shorter intervals linked to better outcomes—highlighting the clinical importance of timely surgical intervention. In the clinical context of a young patient usually a drug user with infection of left sided heart valves with a clot of high likelihood to embolize to the brain, early surgery even after a stroke is associated with improved in-hospital mortality. Delay in such patients increases death from endocarditis.

#### 3.2.2 Benchmarking against traditional feature engineering

The consistent performance of various LLMs suggests that their embeddings effectively capture core data concepts, offering a reliable framework for benchmarking model interpretability. Deviations in future experiments may signal either deeper conceptual understanding or limitations in capturing key patterns—highlighting the influence of proprietary model weights versus embeddings.

This consistency across architectures indicates that LLM embeddings generalize well, regardless of training objectives. In more complex datasets, such as unstructured clinical notes, performance differences may become more pronounced. This framework can then help identify which models best capture meaningful data aspects, using both predictive metrics (e.g., AUC) and conceptual alignment (e.g., SHAP feature similarity).

### 3.3 Clinical applications

#### 3.3.1 Empirical validation of clinical relevance

Supplementary Figure S9 presents SHAP scores from the LLM-unassisted model, with postoperative ejection fraction (EF) emerging as the strongest predictor of hospital mortality. Lower EF values (indicated by red SHAP points) correlate with increased mortality risk, consistent with clinical understanding that impaired cardiac function elevates surgical risk (Topkara et al., 2005; Kim et al., 2024).

Additional variables reinforce known clinical patterns. Female gender appears protective, with lower SHAP values (blue points clustered near zero), while male gender is associated with slightly higher mortality risk. Older age is associated with an increased risk, as indicated by the red points on the positive SHAP axis. Extremes in body mass index (BMI)—both underweight and obese—also contribute to poor outcomes. A history of cardiac arrest or intraoperative arrest significantly raises mortality risk (Fielding-Singh et al., 2020), reflected in high SHAP contributions. Larger vegetations (>1 cm) predict worse outcomes due to increased embolic risk and procedural complexity. “Days from stroke to surgery” is a critical variable: longer delays (red, high feature values) are associated with higher mortality, while shorter delays (blue) may be neutral or protective, suggesting earlier intervention improves outcomes. Selection bias cannot be excluded here that is to say the healthier patients underwent surgery earlier and had better outcomes.

Supplementary Figure S10 shows SHAP scores from the LLM-assisted model, highlighting intravenous drug use (IVDU) as a significant predictor of hospital mortality in endocarditis patients (Tan et al., 2020; Citro et al., 2022; Nguemeni Tiako et al., 2019). This aligns with evidence linking IVDU to tricuspid valve endocarditis (Stolear et al., 2024; Pietrolungo and Gandler, 2023), often complicated by septic pulmonary emboli (SPEs). SHAP plots for SPEs show moderate contributions to mortality risk, with red points (high feature values) indicating higher risk and blue points (low feature values) suggesting minimal impact. This separation underscores SPEs as clinically meaningful indicators of disease severity.

These findings suggest that LLM-assisted models can integrate detailed, clinically relevant information, potentially enhancing predictive performance and interpretability in complex cases.

#### 3.3.2 Real-world applicability

SHAP plots from LLM-assisted models [e.g., E5-small, DistilBERT, GatorTron (Wu, 2023; Akkur, 2023)] reveal consistent influence from core features like age, BMI, and preoperative EF, while each model emphasizes different clinical variables—GatorTron highlights IVDU and catheterization, DistilBERT focuses on surgical and valvular conditions. These differences suggest that each LLM captures distinct aspects of patient profiles, which can be leveraged for subpopulation-specific modeling and personalized care. One-hot encoded features support precise stratification, helping clinicians select models that best reflect disease mechanisms. Patient-specific predictions visualized using tools like LIME, help bridge statistical outputs and clinical interpretability. As shown in Supplementary Figure S11, a case from the high-risk tertile of the endocarditis dataset illustrates a 75% survival prediction for a 28-year-old male, emphasizing the importance of completing antibiotic therapy. Such visualizations enhance clinician-patient communication and support shared decision-making by clarifying how individual variables influence outcomes.

### 3.4 Strengths

This study shows that LLMs with lower computational demands can perform comparably to more resource-intensive models using geometric metrics, making them suitable for deployment on modest hardware. Synthetic data enabled pre-deployment testing across varying sample-to-variable ratios and class imbalances, improving efficiency. LLM-assisted models, combined with interpretability tools like SHAP and LIME, produced clinically meaningful insights in real-world datasets and are scalable to larger applications. The methodology is adaptable across healthcare domains, with clustering and embedding techniques revealing actionable patterns. Deployment guidelines include using de-identified data locally, managing platform licensing, and ensuring secure environments. Interpretability was enhanced through dimensionality reduction techniques, and benchmarking on structured data provided a novel way to assess model alignment and feature relevance.

A key strength of LLMs lies in their ability to leverage attention mechanisms to identify clinically relevant information within complex inputs. Through the Query–Key–Value framework, the model calculates similarity scores between the query and each key, applies scaling and softmax normalization, and generates a weighted sum that privileges the most contextually important features. This enables the model to process nuanced clinical questions—for example, estimating the likelihood of tricuspid valve endocarditis in a pregnant patient with substance use—without reducing them to binary classifications. Instead, the model distributes probabilities across potential interpretations, reflecting the inherent uncertainty of real-world clinical reasoning.

LLMs also integrate probabilistic reasoning akin to Bayesian inference, allowing them to operate effectively under conditions of incomplete, noisy, or uncertain data. In this framework, attention determines which components of the clinical presentation warrant emphasis, while probabilistic inference estimates the degree of certainty associated with a conclusion. Such reasoning parallels the implicit cognitive processes used by experienced clinicians and provides a systematic means of scaling these insights across populations and settings.

A further strength lies in the high-dimensional embedding space through which LLMs represent knowledge. Rather than storing discrete facts, LLMs encode related clinical concepts as clustered vectors in latent space. This facilitates recognition of patterns such as the relationships between endocarditis, intravenous drug use, pregnancy-related physiological changes, and in-hospital mortality risk. In this manner, LLM-assisted analysis can support pattern discovery, risk stratification, and clinical decision-making. Moreover, just as large models can be distilled into smaller, deployable versions, clinician expertise can be analogously aggregated and transmitted via model-guided processes, offering a scalable mechanism to disseminate experiential knowledge.

### 3.5 Limitations

Several limitations warrant consideration. First, evaluating transformer-based models remains challenging due to a lack of consensus on optimal performance metrics. Traditional ML metrics, geometric measures in embedding space, and task-specific clinical validity assessments each capture different properties, and no unified framework currently exists.

Second, constraints related to pretraining data introduce uncertainty. While proprietary clinical datasets and synthetic endocarditis data were excluded from pretraining, complete assurance regarding the presence or absence of publicly available datasets, such as those from the UCI repository, cannot be guaranteed. This complicates claims regarding model naïveté or independence from training exposure.

Computational demands constitute an additional limitation. Training and deploying LLMs require substantial hardware resources and cloud-based infrastructure, contributing to high costs and limiting accessibility for smaller institutions, particularly in rural or resource-limited healthcare systems. Introduction of new hardware designed for inference will likely mitigate inference issues.

Methodologically, preprocessing steps may introduce bias that affect clustering reliability, and the regional origin of the dataset may impose demographic or epidemiologic skew. Such limitations can restrict generalizability beyond the study population. Technical challenges also emerged in modeling efforts, including instability in support of vector regression (SVR) following one-hot encoding, which expanded the feature space and complicated optimization.

Integrating unstructured clinical data, such as provider notes, imaging reports, and free-text histories, remains a major area for future work. Implicit issues in such data include use of templates and autofill in clinical charts resulting in large amounts of copy forward and duplicate data of less clincal relevance with a few sentences devoted to the key clinical issue at hand. Annotation complexity and interpretive variability limit comprehensive incorporation of these high-value data sources.

Finally, although synthetic datasets allowed systematic evaluation and were accompanied by rigorous diagnostic assessments (e.g., residual analyses to assess model assumptions), they cannot fully replicate the complex noise structures, heteroscedasticity, and irregular error patterns found in real-world clinical data. Consequently, model behavior observed in synthetic environments may overestimate robustness when applied to authentic clinical settings.

### 3.6 Conclusion

This study highlights the transformative potential of integrating LLMs with machine learning for clinical data analysis. Using geometric evaluation and interpretability tools like SHAP and LIME, we show that even resource-efficient models can match or outperform more complex architectures. This paves the way for scalable, cost-effective, and privacy-preserving healthcare, especially when synthetic data protects patient confidentiality.

However, challenges remain. Variability in model performance, computational demands, and demographic biases limit generalizability. SVR constraints further complicate modeling in high-dimensional, categorical-rich datasets. Future work integrating unstructured clinical text could enhance model depth and interpretability, bridging structured and unstructured data to advance precision medicine through transparent, adaptable, and ethically grounded AI.

## Data Availability

The original contributions presented in the study are included in the article/Supplementary material, further inquiries can be directed to the corresponding author.
