# Supplementary figures and images for: Unveiling patterns in clinical data: exploring the role of large language models and clustering algorithms
Source: Front Artif Intell. 2026 Mar 9;9:1737530. doi: 10.3389/frai.2026.1737530 (PMC13006407; doi:10.3389/frai.2026.1737530)

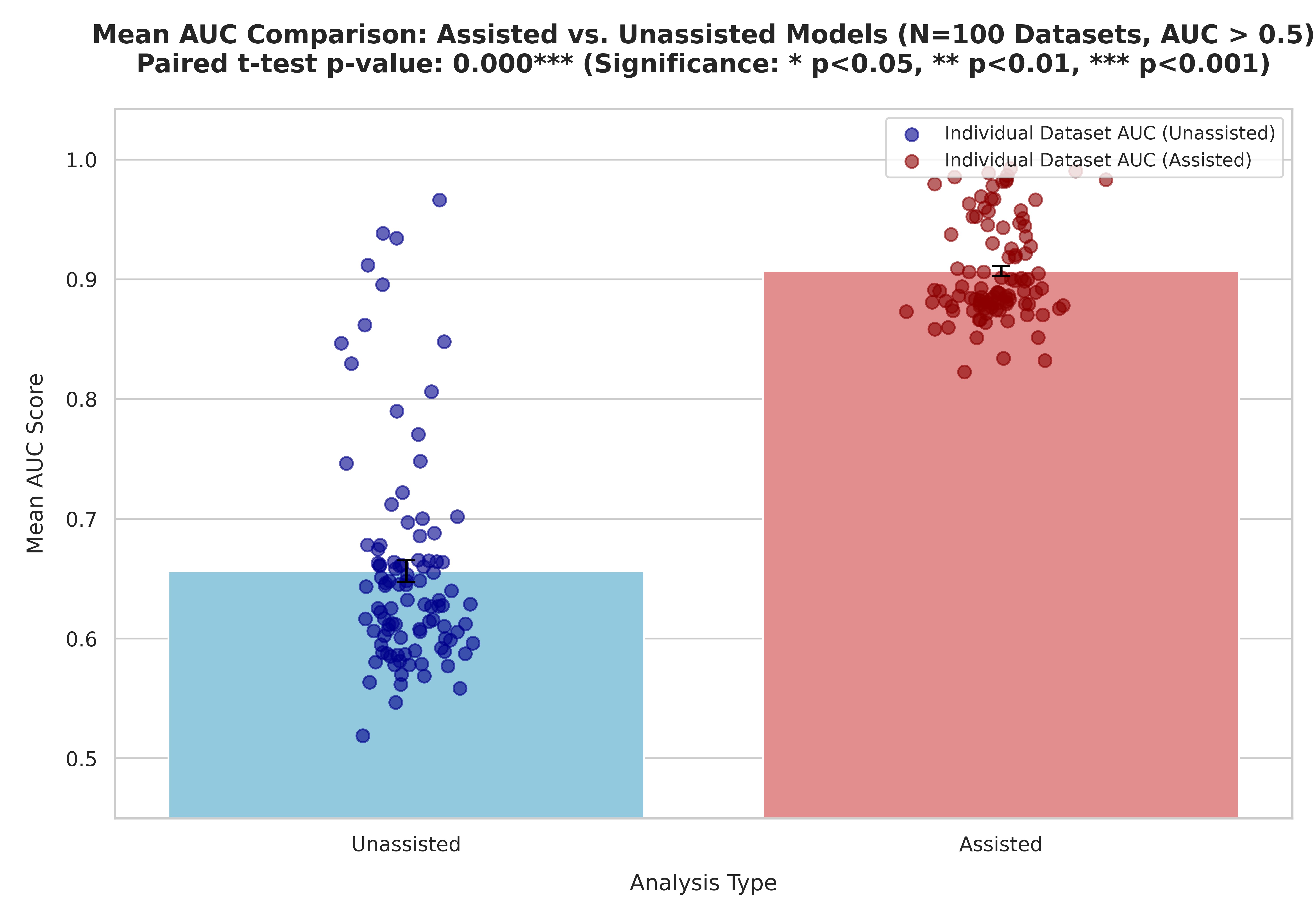

Supplement: Supplementary file 1 [file Supplementary_file_1.zip › SuppFig3.jpg]

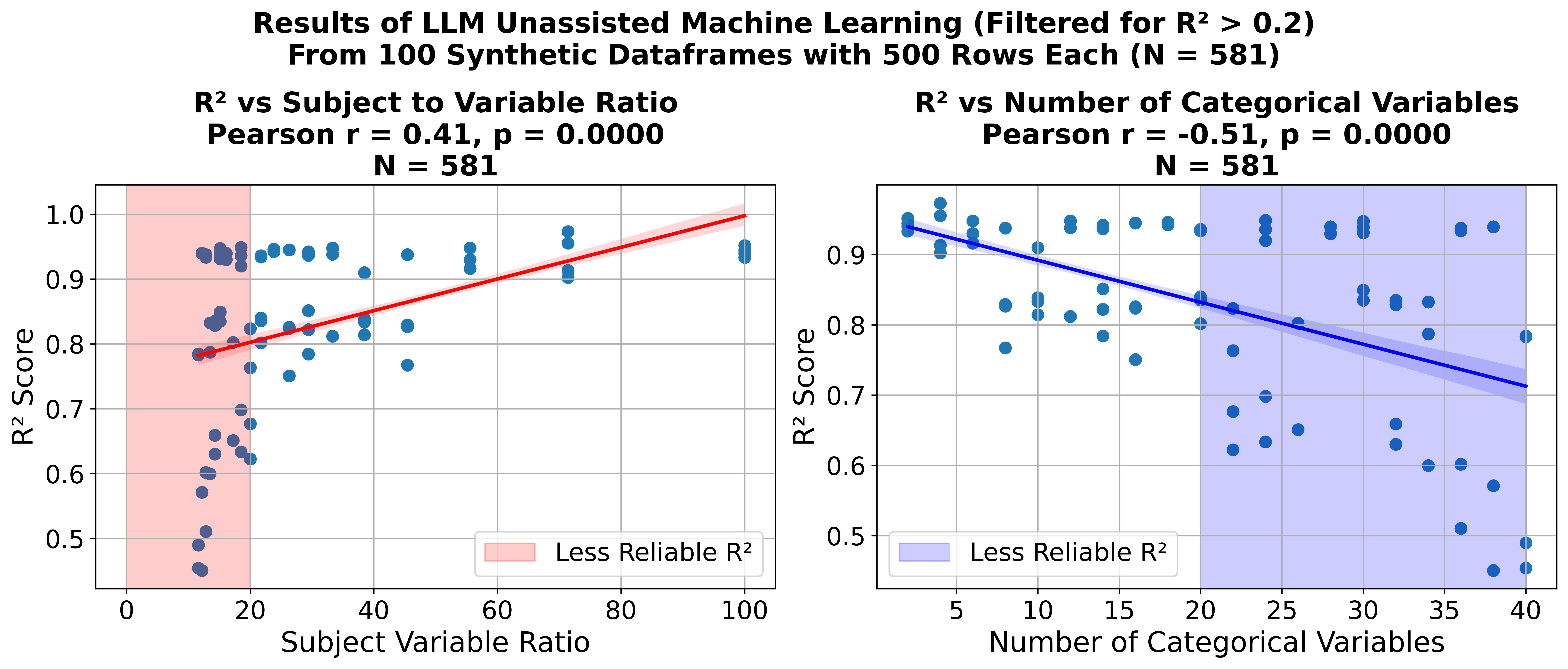

Supplement: Supplementary file 1 [file Supplementary_file_1.zip › SuppFig4.jpg]

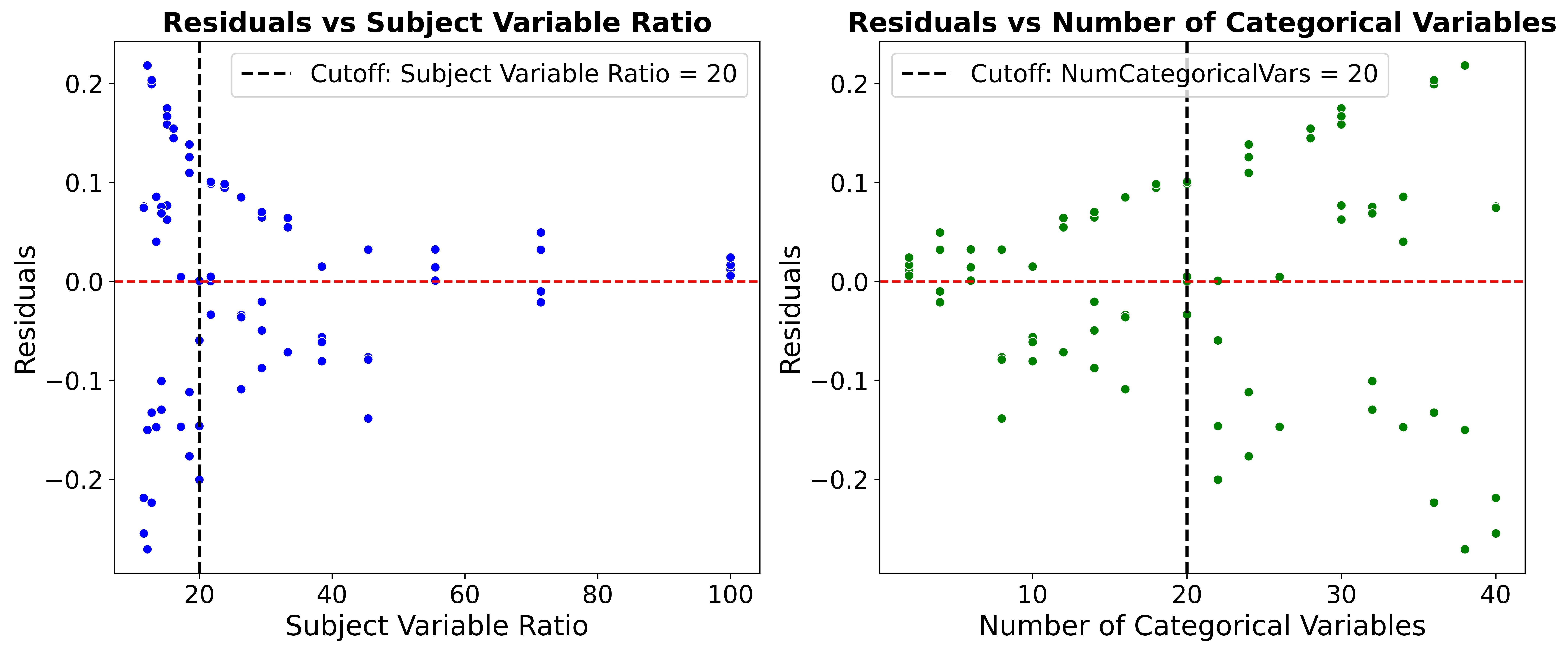

Supplement: Supplementary file 1 [file Supplementary_file_1.zip › SuppFig5.jpg]

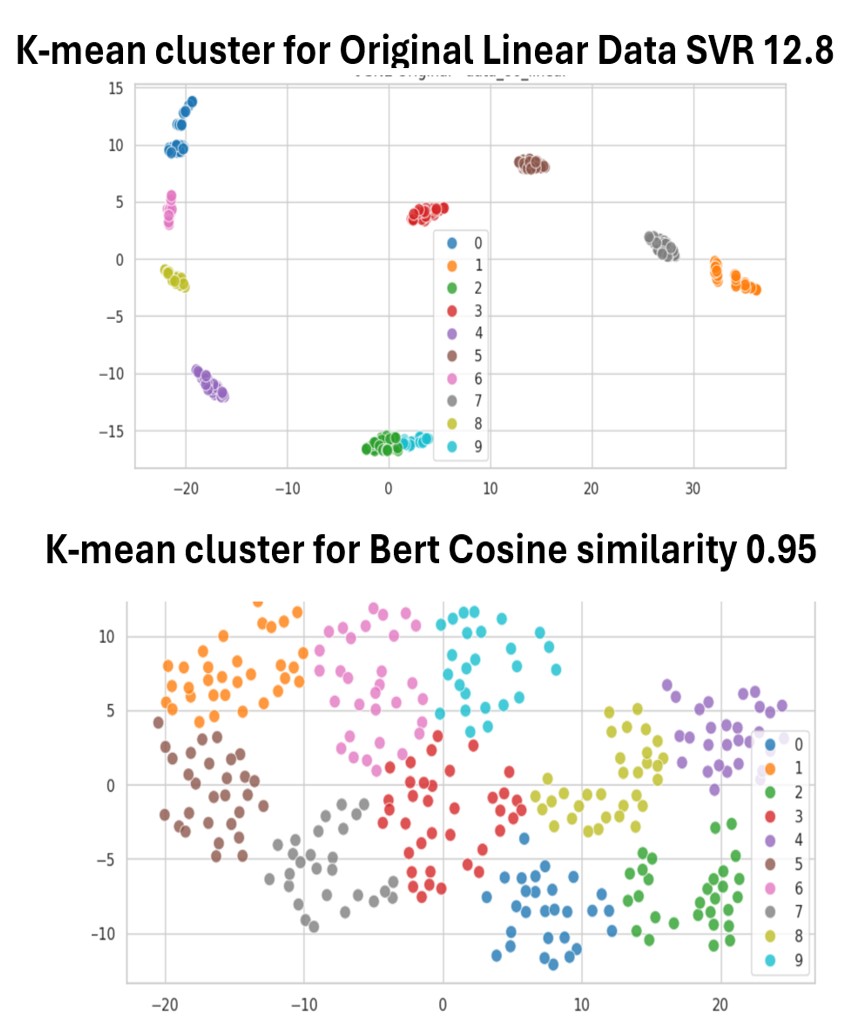

Supplement: Supplementary file 1 [file Supplementary_file_1.zip › SuppFig6.jpg]

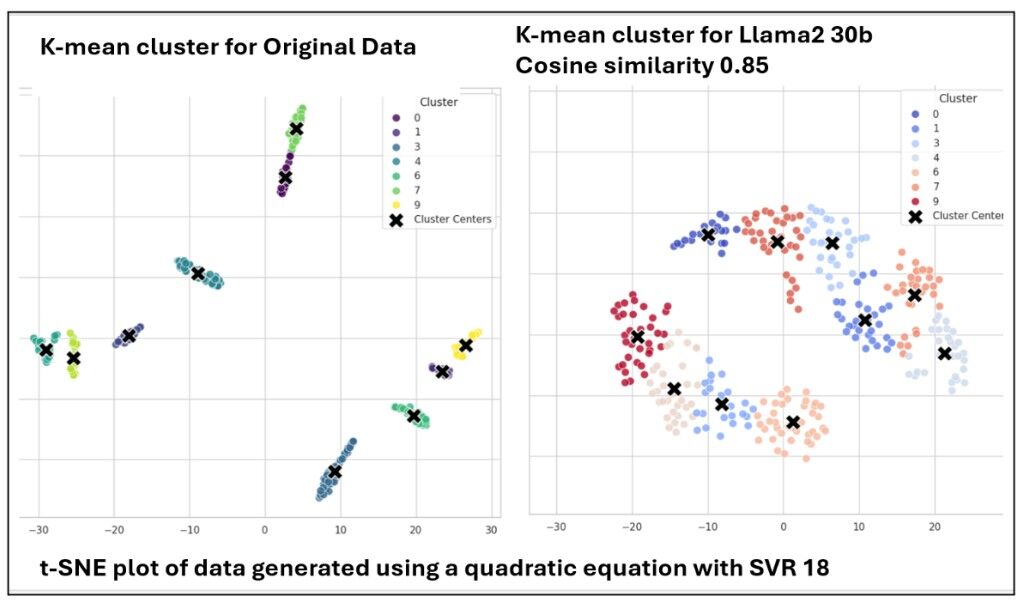

Supplement: Supplementary file 1 [file Supplementary_file_1.zip › SuppFig7.jpg]

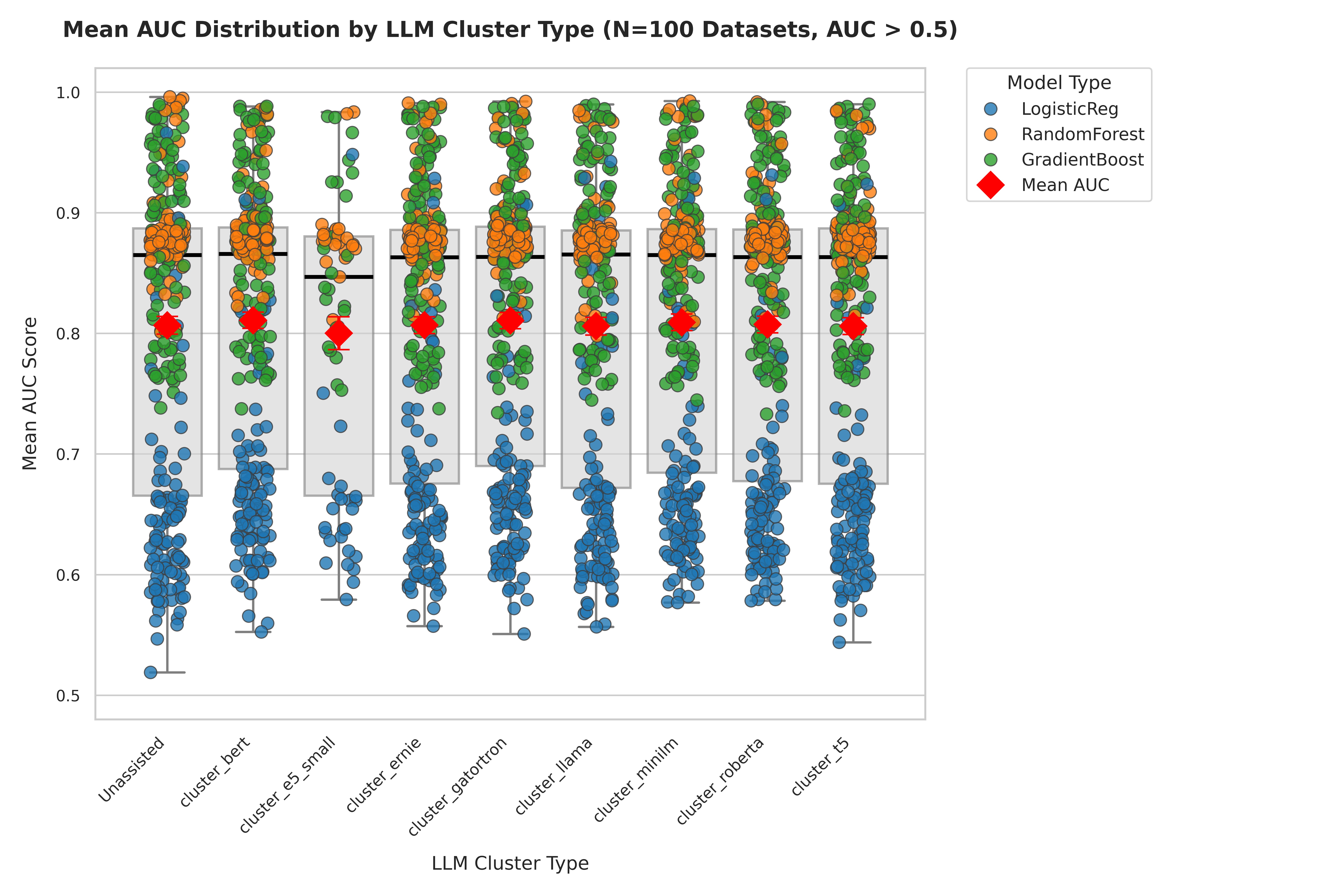

Supplement: Supplementary file 1 [file Supplementary_file_1.zip › SuppFig8.jpg]

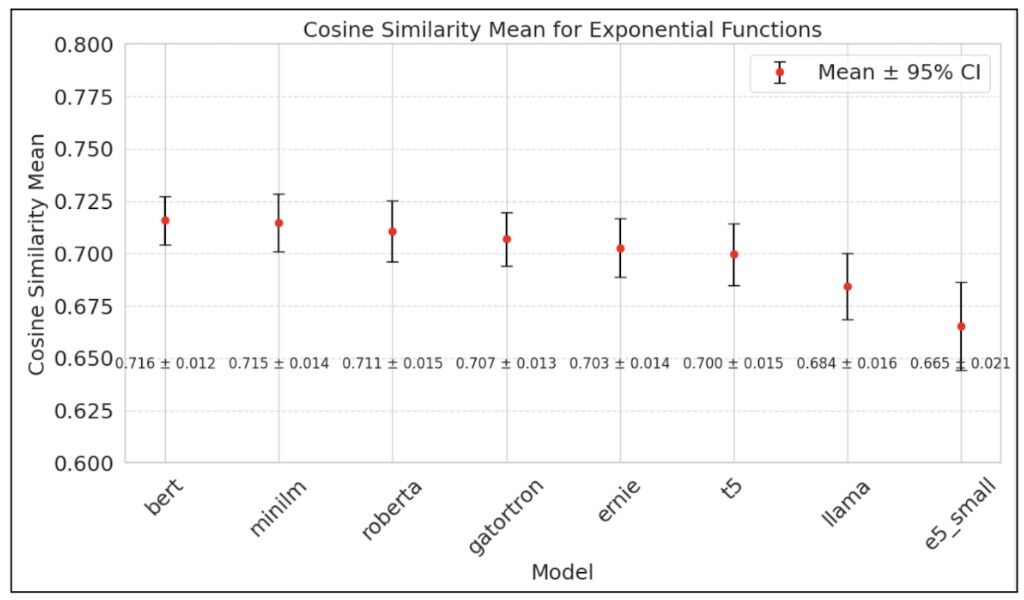

Supplement: Supplementary file 1 [file Supplementary_file_1.zip › SuppFig9.jpg]

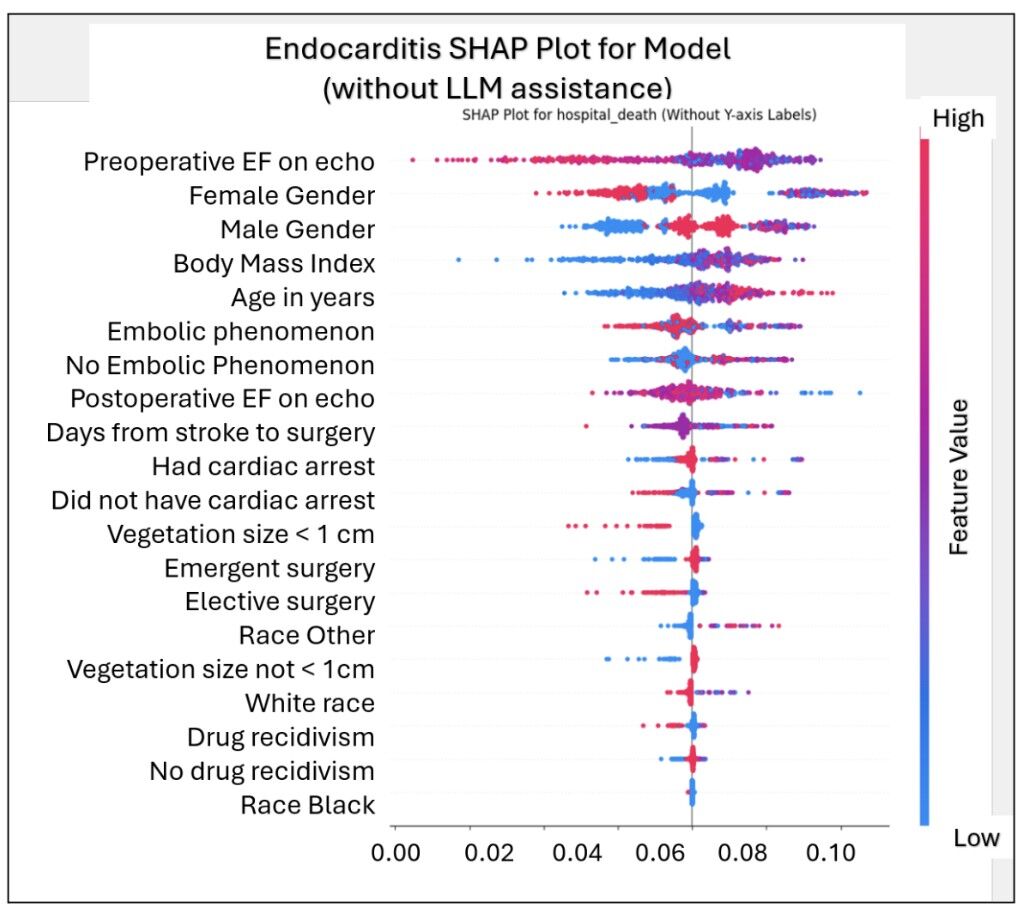

Supplement: Supplementary file 1 [file Supplementary_file_1.zip › SuppFig10.jpg]

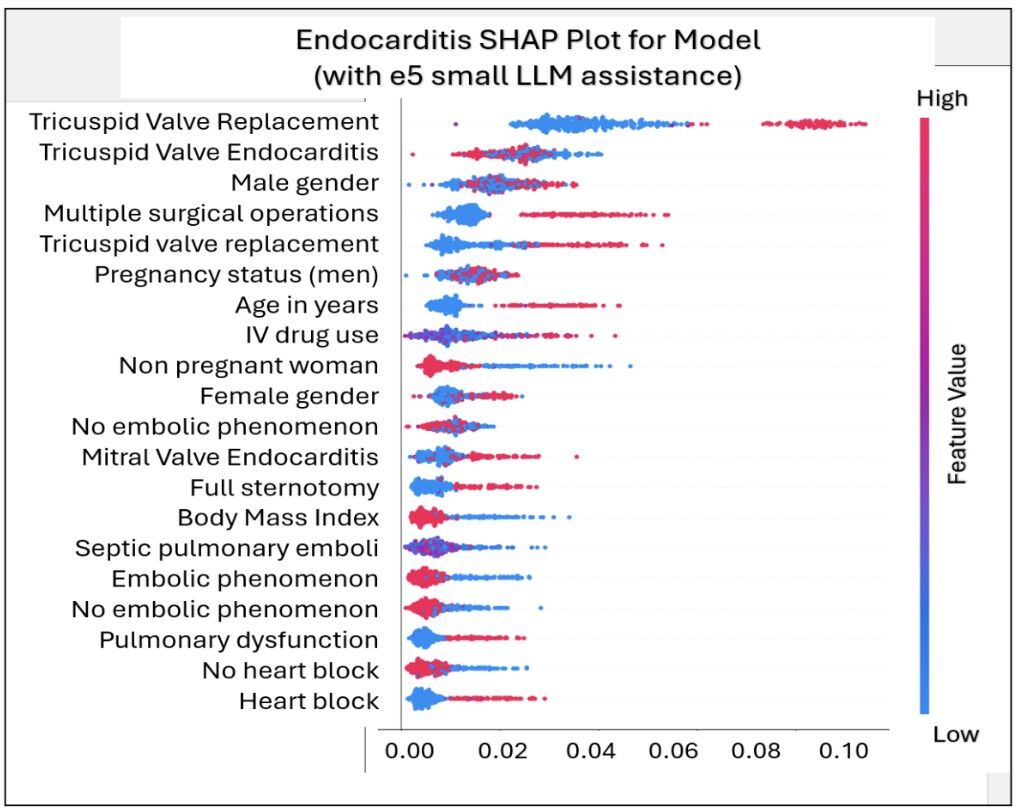

Supplement: Supplementary file 1 [file Supplementary_file_1.zip › SuppFig11.jpg]

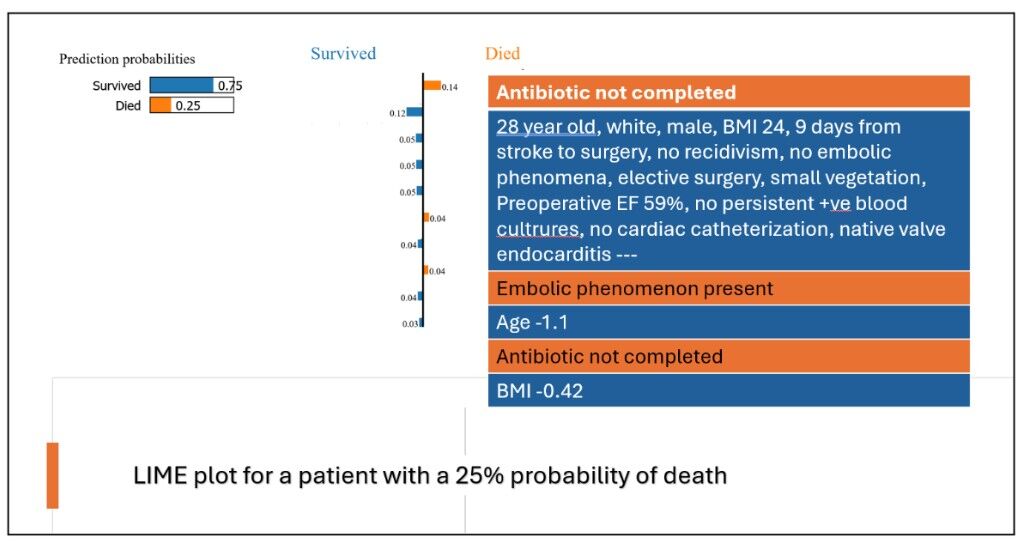

Supplement: Supplementary file 1 [file Supplementary_file_1.zip › SuppFig12.jpg]

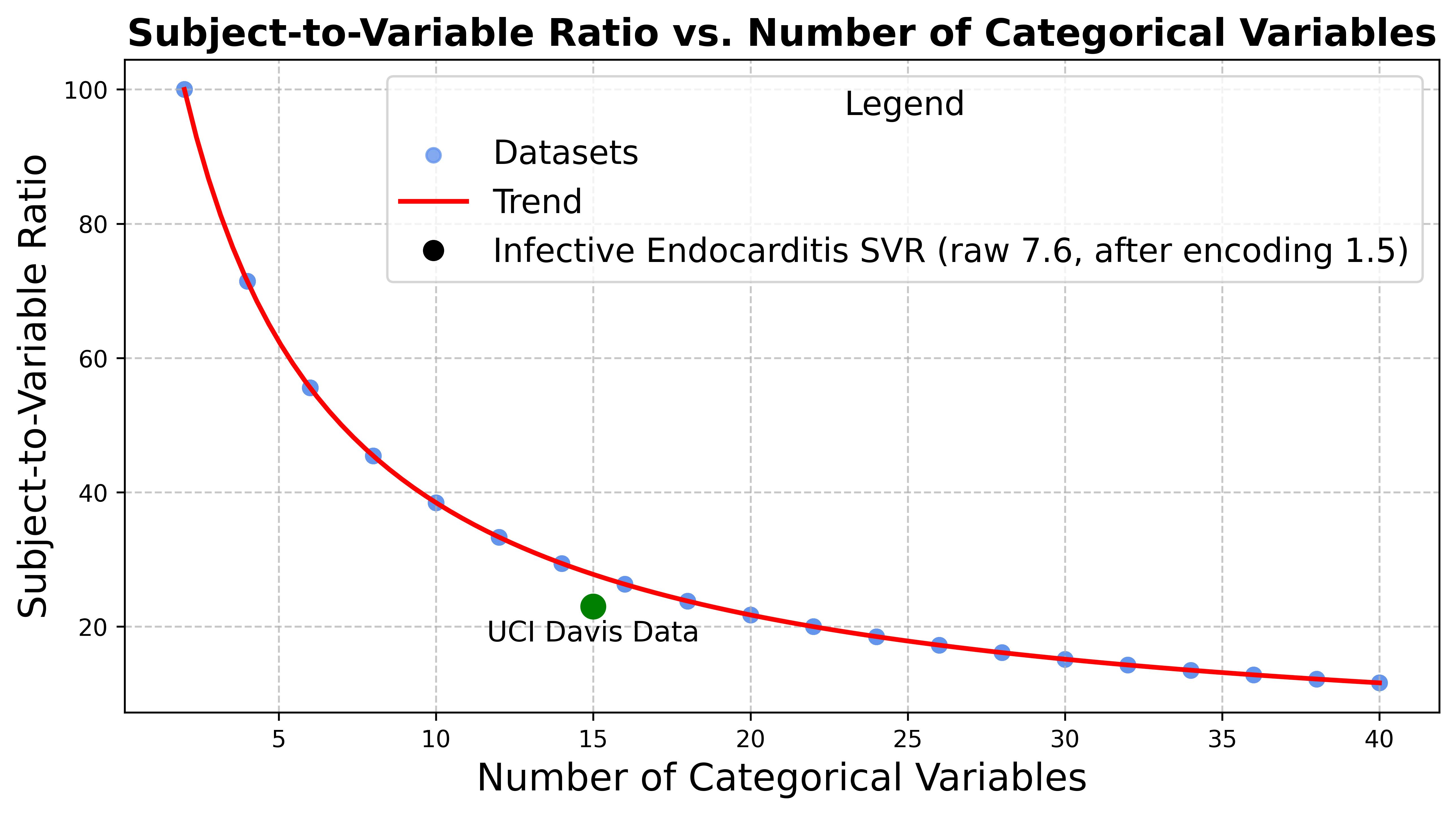

Supplement: Supplementary file 1 [file Supplementary_file_1.zip › SuppFig1.jpg]

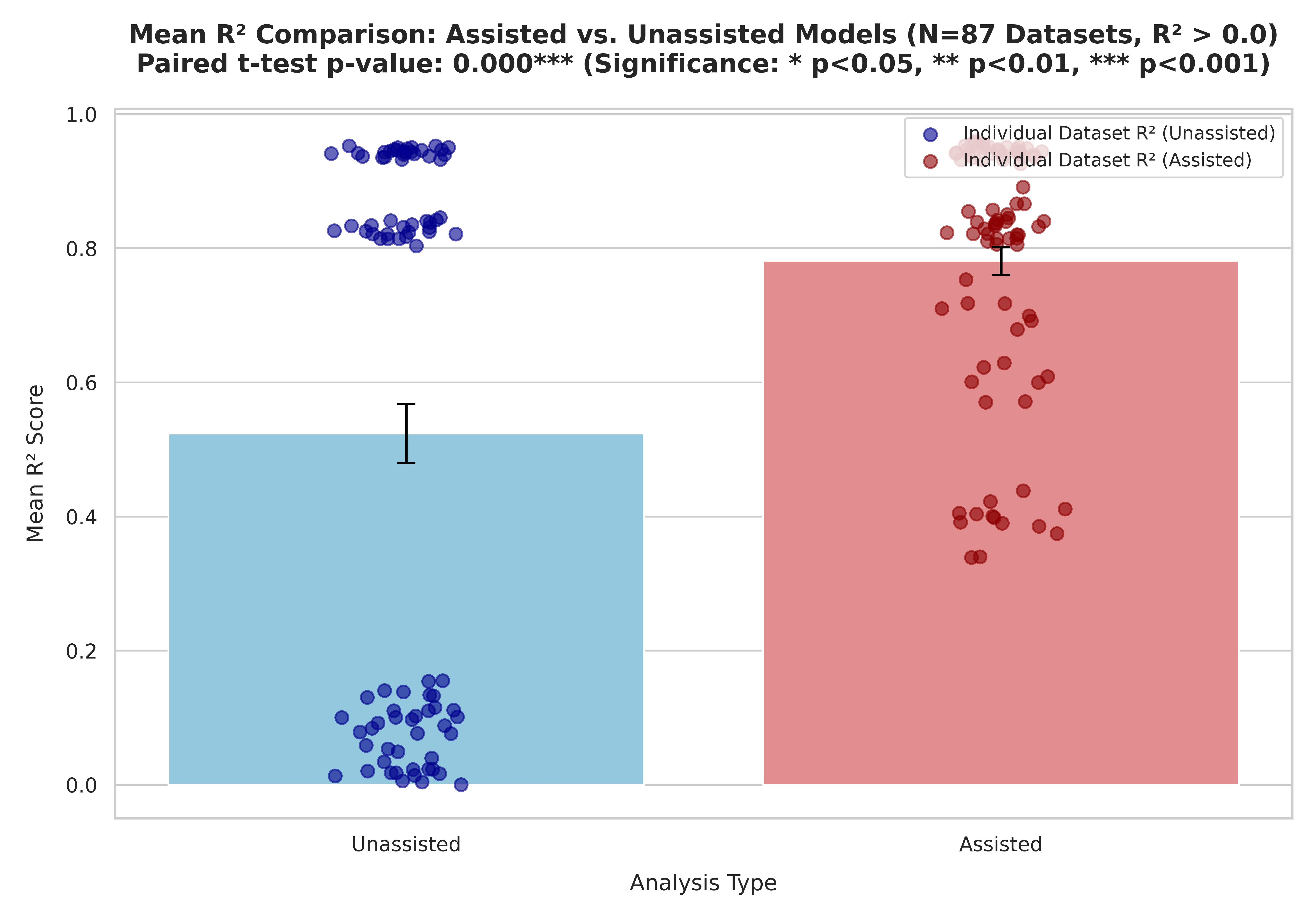

Supplement: Supplementary file 1 [file Supplementary_file_1.zip › SuppFig2.jpg]
